# Responsiveness of five condition-specific and generic outcome assessment instruments for chronic pain

**DOI:** 10.1186/1471-2288-8-26

**Published:** 2008-04-25

**Authors:** Felix Angst, Martin L Verra, Susanne Lehmann, André Aeschlimann

**Affiliations:** 1 Research department, Rehaclinic Zurzach, Bad Zurzach, Switzerland; 2 Department of Epidemiology and Caphri Research Institute, Maastricht University, Maastricht, The Netherlands

## Abstract

**Background:**

Changes of health and quality-of-life in chronic conditions are mostly small and require specific and sensitive instruments. The aim of this study was to determine and compare responsiveness, i.e. the sensitivity to change of five outcome instruments for effect measurement in chronic pain.

**Methods:**

In a prospective cohort study, 273 chronic pain patients were assessed on the Numeric Rating Scale (NRS) for pain, the Short Form 36 (SF-36), the Multidimensional Pain Inventory (MPI), the Hospital Anxiety and Depression Scale (HADS), and the Coping Strategies Questionnaire (CSQ). Responsiveness was quantified by effect size (ES) and standardized response mean (SRM) before and after a four week in-patient interdisciplinary pain program and compared by the modified Jacknife test.

**Results:**

The MPI measured pain more responsively than the SF-36 (ES: 0.85 vs 0.72, p = 0.053; SRM: 0.72 vs 0.60, p = 0.027) and the pain NRS (ES: 0.85 vs 0.62, p < 0.001; SRM: 0.72 vs 0.57, p = 0.001). Similar results were found for the dimensions of role and social interference with pain. Comparison in function was limited due to divergent constructs. The responsiveness of the MPI and the SF-36 was equal for affective health but both were better than the HADS (e.g. MPI vs HADS depression: ES: 0.61 vs 0.43, p = 0.001; SF-36 vs HADS depression: ES: 0.54 vs 0.43, p = 0.004). In the "ability to control pain" coping dimension, the MPI was more responsive than the CSQ (ES: 0.46 vs 0.30, p = 0.011).

**Conclusion:**

The MPI was most responsive in all comparable domains followed by the SF-36. The pain-specific MPI and the generic SF-36 can be recommended for comprehensive and specific bio-psycho-social effect measurement of health and quality-of-life in chronic pain.

## Background

Chronic pain is a syndrome of multiple etiology and has consequences for somatic, psychological and psycho-social well-being, functionality and health related quality-of-life [1]. Outcome assessment of chronic pain should comprehensively cover all relevant dimensions of these health characteristics and should, therefore, be performed with generic measurement tools [2,3]. However, more comprehensive measurement is often tied up with less sensitive assessment in specific domains as shown in various studies: In the assessment of shoulder arthritis, a dose-response curve of specificity and responsiveness could be empirically proven [4].

Improvements following interventions for chronic pain disorders are often small and their detection requires specific instruments which are sensitive to change, i.e. responsive [5-7]. Responsiveness is, therefore, besides reliability and other aspects of validity, one of the most important properties of an outcome measure [8]. It is the basis on which the 'discrimination' criteria were established by the quality classification process of the Outcome Measures in Rheumatology Clinical Trials (OMERACT) carried out by the World Health Organization (WHO), the American College of Rheumatology (ACR), and the European League Against Rheumatism (EULAR) [9].

There are several methods to measure responsiveness. Commonly used is the effect size (ES) which gives a continuous parametric measure of the change between baseline and follow-up and can be easily interpreted – determination and interpretation: see in Methods [8,10-13]. However, many reports used the standardized response mean (SRM) which often results in similar values (see in Results) as the ES – determination and interpretation: see in Methods [14]. In this study, we reported both parameters to be comparable to the majority of findings in the literature. A similar parameter is provided by the Guyatt's responsiveness statistics but its determination requires a two point measurement of a "stable" time period, i.e. without health change and interventions and is, therefore, often not available [15,16]. It often results in higher values than the ES and the SRM as can be seen in the comparison of all three parameters in [7]; see also [11]. In case an external criterion (e.g. improved versus unchanged) or a diagnostic threshold of a score (e.g. score ≥ 60 for severe depression) is known as "gold" standard or "anchor" the receiver operating characteristics (ROC) curve is a sensitive method to characterize responsiveness. It provides sensitivity, specificity, negative and positive predictive values, and the area under ROC gives a goodness of fit measure of a test [13,14,16,17]. Further advantages and disadvantages of the different methods can be found in the indicated references.

Comparison of the responsiveness of two scales only makes sense if they measure more or less the same content and construct within the same domain, e.g. in pain, function or affective health [18,19]. This means that the two scales should have a high construct overlap which is most often quantified by the correlation between the two scales [19,20]. To our knowledge, only one study exists examining and comparing the responsiveness of different self-assessment instruments in chronic pain [19].

The present study aimed to determine and compare the responsiveness of five self-assessment instruments widely used in the evaluation of chronic pain patients in an effort to identify the best instruments and scales for the measurement of specific health and quality-of-life dimensions. We hypothesized that a condition-specific instrument is more responsive than a generic one.

## Methods

### Patients

The subjects included in the study were all participants of the "Zurzach Interdisciplinary Pain Program" (ZISP) who were suffering either from chronic non-specific back pain (i.e. lumbar, thoracic, cervical, or panvertebral pain syndrome), or fibromyalgia according to the definition of the American College of Rheumatology (ACR), or chronic widespread pain, i.e. generalized musculoskeletal pain syndrome which did not meet the definition criteria of fibromyalgia [21]. The ZISP program is a comprehensive, standardized, four week inpatient pain program at the rehabilitation clinic "RehaClinic", Bad Zurzach, Switzerland and consists mainly of medical care including adapted drug therapy, graded activity exercise, and cognitive behavioral therapy. A detailed description of the program with the inclusion and exclusion criteria has already been published as part of our outcome paper [5].

### Measures

The Short Form 36 (SF-36) is the most widely accepted and frequently used generic instrument that comprehensively measures physical, mental and psychosocial health by means of 36 items (questions) that determine 8 scales [22,23]. The West Haven-Yale Multidimensional Pain Inventory (WHYMPI, abbreviated to MPI) assesses pain and pain-specific consequences in terms of symptoms, activity, behavior, mood, and social relationships on the basis of 51 items that construct 12 scales [24,25]. The Hospital Anxiety and Depression Scale (HADS) measures anxiety and depression based on 7 items each and is well established in psychology and psychiatry with a long history of application [26,27]. The Coping Strategies Questionnaire (CSQ) is the tool most often used to assess cognitive and behavioural strategies to tolerate, manage and compensate for pain and their consequences, and is based on 48 items resulting in 8 scales plus 2 additional control items [28-30]. All four instruments are standardized, well tested and widely used – a quick search in MedLine showed 2000–6300 citations for each of the four tools (February 26, 2008). In addition, current pain was assessed by the 11-point Numeric Rating Scale (NRS) ranging from 0 = no pain to 10 = most pain imaginable [31].

On scale level, two instruments can be compared if the items that make up the scales ask about the same domain, i.e. have the same construct [19,20]. Thus, MPI pain severity was compared with the SF-36 bodily pain and the pain NRS for the assessment of pain. SF-36 role physical together with SF-36 social functioning were compared to MPI interference with pain in the assessment of somatic and psychosocial consequences of the pain disorder covering activities of daily living, work, leisure, and social participation. Function, including ambulation and specific activities (home and outdoor), was covered by SF-36 physical functioning and MPI general activities score; the latter was determined by all 18 activity items as previously described [32]. Affective health/mood (explicitly: happiness, tension, irritability, nervousness, calmness/quietism) was assessed by HADS depression and anxiety scales and compared to SF-36 mental health and MPI negative mood. Control over pain was measured by MPI control pain and CSQ control pain (each by one item). These domains have been previously described and the overlap of their constructs has been tested empirically [19].

### Analysis

Assessments were performed at entry into the clinic (baseline) and in the last two days before discharge, i.e. four weeks after entry (follow-up). The scores were determined following the "missing rules" of the instruments, i.e. to determine a score, at least 50% of the items had to be filled out for the SF-36 and 6/7 (86%) for the HADS [22,27]. For the MPI and the CSQ, where the developers of the questionnaires do not describe missing rules, we used the previously described 2/3 (67%) criteria [5,6,30]. The score range was transformed into 0 = maximal pain/no function/worst coping/worst health to 100 = no pain/full function/best coping/best health for all instruments' scores as originally described for the SF-36 to ease comparison between them with the exception of the pain NRS (0 = no pain, 10 = maximal pain) [5,6,22]. All analyses were performed using the statistical software package SPSS 16.0 for Windows^® ^(SPSS Inc., Chicago, IL, USA).

The score difference between follow-up and baseline divided by the standard deviation of the group's baseline scores is defined as effect size (ES), originally introduced as "Glass's delta" [10,12]. The score difference (follow-up – baseline) divided by the standard deviation of the group's score differences determines the standardized response mean (SRM), originally published as the "Hedge's g" for one sample which is equal to the "Cohen's delta" in this case [12,14]. The ES and the SRM are the most common measures for responsiveness. Positive values reflect (standardized) improvements in the number of standard deviations of the baseline scores (ES) or the score differences (SRM) (i.e. unit-free) [7,11]. An ES ≥ 0.80 is considered as large, 0.50–0.79 as moderate, 0.20–0.49 as small, and 0.00–0.19 as very small [10].

To test whether the difference of two responsiveness measures within a certain domain was statistically significant, the "modified Jacknife test" was applied [7,18]. This method is a linear regression between the difference of the ES or SRM of two comparable scores (e.g. between SF-36 bodily pain and MPI pain severity) as dependent variable and the "centered" ES/SRM of one of the two scales (which scale is not relevant) as independent variable. If the regression's intercept (value of the SRM/ES difference where the centered ES/SRM is equal to zero) is greater or smaller to zero with significance p < 0.050 there is significant difference of the responsiveness of the two scales. For that, the difference of the two ES/SRM are computed in SPSS individually for each patient as well as the "centered" ES/SRM which is equal to the individual ES/SRM minus the (mean) ES/SRM of the whole sample [18].

In multiple pairwise testing of (at least partly) non independent scores (e.g. within the patient-rating of pain), the significance level must be reduced by the number of tested scores (k), i.e. p = 0.05/(k!/(k-2)!*2!) which is well know as the Bonferroni-correction [33]. Thus, the significance level for type I error was set at p = 0.050/3 = 0.017 for comparison of k = 3 instruments (MPI, SF-36, pain NRS in pain and SF-36, MPI, HADS in affective health) and at p = 0.050 for comparison of two instruments.

To quantify the extent of the overlapping constructs within a domain, bivariate Spearman rank correlation coefficients of the baseline scores and the effects (raw score differences baseline → follow-up) were determined for each pair of scales being compared [20].

An additional way to assess the size of effects is to compare the ES with the minimal important difference (MID) for which the estimate is based on the standard error of measurement (SEM) [34]. The SEM in score units is equal to the baseline standard deviation of the scores multiplied by the square root of (1-r), where r is the reliability measure of the scale, usually the intraclass correlation coefficient [34]. The SEM in responsiveness units is therefore equal to the square root of (1-r) for the ES and equal to the baseline standard deviation divided by the standard deviation of the score differences times the square root of (1-r) for the SRM. Note that the SEM in ES or SRM units is independent of the frequency distribution of the scores, i.e. from the sample itself – it is only dependent on the reliability coefficient. We chose the "one-SEM" criterion which means that 1*SEM is an estimate of the MID, the effect which patients (on average) perceive as subjective change [34]. The MID can be used as an estimate for the minimal clinically important difference (MCID) which principally is an anchor-based (on an external criterion) method to assess the smallest effect that patients perceive to be beneficial. As empirically shown, the MID and the MCID often are in the same size [34] or the MCID is even smaller than the MID (see example in the Discussion: [35]). Further information about the importance of the MCID can be found elsewhere [8,16]. However, the present study compared within-subject and not between-subject effects.

## Results

### Patients

The cohort consisted of 273 chronic pain patients assessed between 1999 and 2006 whose characteristics have been reported in detail elsewhere [5,36]. The median pain duration was 60 months (5 years). The mean age was 46.3 years (standard deviation = 10.5, normally distributed) and 79.9% were female. Fibromyalgia was present in 43.2%, chronic back pain in 42.5%, and chronic widespread pain in 14.3% of the cases. There were very few omissions, i.e. complete data pairs (i.e. 273 score pairs baseline – follow-up) were available for most of the scores: the MPI negative mood and the pain NRS had 272, the HADS anxiety 271, the MPI activity and the MPI control each 270, and the CSQ control 267 complete data pairs.

### Responsiveness analysis

The descriptive data of all scores at baseline and follow-up together with the ES and the SRM, and the MCID are shown in Table 1. We report all data of the instruments for completeness. Effects for SF-36 vitality and MPI pain severity showed great improvement (ES≥ 0.80). Moderate effects, i.e. ES between 0.50 and 0.79 were recorded for the pain NRS, SF-36 role physical, SF-36 bodily pain, SF-36 social functioning, SF-36 mental health, SF-36 mental component summary (MCS), MPI interference with pain, MPI life control, and MPI negative mood. All other effects were small; those of MPI support and MPI punishing/solicitous/distracting responses scales and 7 of the 10 CSQ scales even very small (ES < 0.20).

**Table 1:** Descriptive and responsiveness data (n = 273)

	**Entry**		**Discharge**		**Difference**		**Sample**	**Sample**	**MCID**	**MCID**	**Reliability**
				
	**m**	**s**	**m**	**s**	**m**	**s**	**ES**	**SRM**	**ES**	**SRM**	**ICC**
**Pain NRS**	6.79	2.09	5.49	2.40	1.30	2.28	0.62	0.57	0.20	0.18	0.96

**SF-36 physical functioning**	39.5	21.0	48.1	22.3	8.5	18.2	0.41	0.47	0.26	0.30	0.93
**SF-36 role physical**	8.1	18.3	19.9	29.0	11.8	29.5	0.65	0.40	0.30	0.19	0.91
**SF-36 bodily pain**	18.1	13.5	27.9	15.7	9.8	16.3	0.72	0.60	0.33	0.27	0.89
**SF-36 general health**	41.1	15.8	45.4	17.1	4.3	14.8	0.27	0.29	0.57	0.61	0.67
**SF-36 vitality**	26.2	16.9	40.5	19.5	14.3	17.9	0.85	0.80	0.37	0.35	0.86
**SF-36 social functioning**	41.6	25.5	55.3	25.3	13.7	25.5	0.54	0.54	0.41	0.41	0.83
**SF-36 role emotional**	31.5	41.5	46.0	44.3	14.5	46.5	0.35	0.31	0.32	0.28	0.90
**SF-36 mental health**	45.7	19.6	56.3	18.2	10.6	16.0	0.54	0.66	0.39	0.47	0.85
**SF-36 PCS**	28.5	6.4	31.3	7.2	2.7	6.5	0.43	0.42	0.28	0.28	0.92
**SF-36 MCS**	36.5	11.6	42.7	11.6	6.3	10.0	0.54	0.63	0.32	0.37	0.90

**MPI pain severity**	23.0	15.0	35.7	18.8	12.7	17.6	0.85	0.72	0.42	0.36	0.82
**MPI interference with pain**	28.3	16.7	39.0	19.1	10.7	16.0	0.64	0.67	0.37	0.39	0.86
**MPI life control**	47.8	20.2	58.9	19.4	11.1	21.3	0.55	0.52	0.53	0.50	0.72
**MPI control pain**	42.4	26.0	54.3	22.9	11.9	26.8	0.46	0.44	0.46	0.44	0.79
**MPI negative mood**	42.3	22.1	55.8	22.5	13.5	20.7	0.61	0.65	0.56	0.59	0.69
**MPI support**	68.3	29.6	66.7	28.1	-1.6	16.6	-0.05	-0.10	0.45	0.80	0.80
**MPI punishing responses**	77.3	23.6	78.6	22.6	1.2	20.0	0.05	0.06	0.40	0.47	0.84
**MPI solicitous responses**	55.9	28.6	56.1	28.4	0.2	17.1	0.01	0.01	0.33	0.55	0.89
**MPI distracting responses**	52.4	28.0	52.6	27.9	0.2	20.1	0.01	0.01	0.62	0.86	0.62
**MPI general activities**	39.9	13.4	41.9	15.7	2.0	9.9	0.15	0.20	0.37	0.51	0.86

**HADS anxiety**	49.9	20.9	57.3	20.4	7.4	16.3	0.35	0.45	0.44	0.56	0.81
**HADS depression**	55.5	20.8	64.5	22.5	9.0	15.1	0.43	0.59	0.33	0.46	0.89

**CSQ diverting attention**	51.6	17.8	52.7	17.5	1.1	14.8	0.06	0.08	0.35	0.42	0.88
**CSQ reinterpreting pain**	27.0	20.6	30.1	20.2	3.1	16.2	0.15	0.19	0.32	0.40	0.90
**CSQ self-statements**	58.9	16.9	59.2	15.5	0.3	14.0	0.02	0.02	0.36	0.44	0.87
**CSQ ignoring pain**	46.5	18.6	47.4	17.9	0.8	15.0	0.05	0.06	0.26	0.33	0.93
**CSQ praying or hoping**	49.8	22.7	53.2	21.3	3.4	12.4	0.15	0.27	0.28	0.52	0.92
**CSQ catastrophizing**	45.8	18.4	52.7	19.1	6.9	14.6	0.38	0.48	0.26	0.33	0.93
**CSQ activity level**	57.3	15.0	59.0	14.5	1.7	12.6	0.11	0.14	0.30	0.36	0.91
**CSQ pain behaviors**	62.6	13.9	61.2	12.8	-1.5	10.0	-0.10	-0.15	0.39	0.54	0.85
**CSQ control pain**	43.9	23.0	50.9	19.6	7.0	23.8	0.30	0.29	0.42	0.41	0.82
**CSQ decrease pain**	37.8	20.2	45.9	18.9	8.1	22.0	0.40	0.37	0.67	0.62	0.55

NRS: Numeric Rating Scale, SF-36: Short Form 36, PCS: Physical Component Summary, MCS: Mental Component Summary, MPI: Multidimensional Pain Inventory, HADS: Hospital Anxiety and Depression Scale, CSQ: Coping Strategies Questionnaire. m: Mean, s: Standard deviation, ES: Effect Size, SRM: Standardized Response Mean. MCID: Minimal Clinically Important Difference in units of the ES or the SRM (see in Methods, Analysis), ICC: Intraclass Correlation Coefficient (obtained by literature review: i.e. the instruments' manuals). Scaling: 0 = no pain, 10 = most pain for the pain NRS; 0 = worst, 100 = best for all instruments' scales

Comparing the ES and SRM data within the same scale, the differences were mostly small (i.e. |ES-SRM| ≤ 0.10) except for SF-36 role physical, SF-36 bodily pain, the SF-36 mental health, SF-36 mental component summary (MCS), MPI pain severity, HADS depression, and CSQ praying or hoping. In SF-36 role physical, the reason for this may be the high floor effect: The scores at baseline were close to zero which resulted in a small baseline standard deviation and, by that, in a high ES when compared to the lower SRM which is determined by the relatively higher standard deviation of the difference. The effects of the sample were slightly or much higher than the estimated MCID (3^rd ^and 2^nd ^columns from the right of Table 1) of the Pain NRS, in 9/10 SF-36 scales, 4/10 MPI scales, 1/2 HADS scales, and 1/10 CSQ scales.

Table 2 shows the comparison between the responsiveness measures of those instruments measuring the same construct domain. The MPI was more responsive than the SF-36 in the effect measurement of pain (and also than the pain NRS), role interference with pain (only by the SRM), and social interference with pain. The MPI was also more responsive than the HADS in affective health (but not better than the SF-36), and more responsive than the CSQ in coping (ability to control pain). In mood assessment the SF-36 was overall more responsive than the HADS.

**Table 2:** Comparison of the responsiveness of the scales within the same construct domains

**Domain**	**Higher responsiveness**	**Lower responsiveness**	**ES**	**p(ES)**	**SRM**	**p(SRM)**	**r base**	**r diff**
**Pain**	MPI pain severity	Pain NRS	0.85 vs 0.62	< 0.001	0.72 vs 0.57	0.001	0.76	0.64
**Pain**	MPI pain severity	SF-36 bodily pain	0.85 vs 0.72	0.053	0.72 vs 0.60	0.027	0.59	0.41
**Pain**	SF-36 bodily pain	Pain NRS	0.72 vs 0.62	0.144	0.60 vs 0.57	0.606	0.52	0.34
**Role interference with pain**	MPI interference with pain	SF-36 role physical	0.64 vs 0.65	0.892	0.67 vs 0.40	< 0.001	0.23	0.27
**Social interference with pain**	MPI interference with pain	SF-36 social functioning	0.64 vs 0.54	0.050	0.67 vs 0.54	0.015	0.50	0.40
**Function**	SF-36 physical functioning	MPI activity	0.41 vs 0.15	< 0.001	0.47 vs 0.20	< 0.001	0.09	0.18
**Affective health: depression**	MPI negative mood	SF-36 mental health	0.61 vs 0.54	0.169	0.65 vs 0.66	0.842	0.73	0.40
**Affective health: depression**	SF-36 mental health	HADS depression	0.54 vs 0.43	0.004	0.66 vs 0.59	0.180	0.73	0.53
**Affective health: depression**	MPI negative mood	HADS depression	0.61 vs 0.43	0.001	0.65 vs 0.59	0.296	0.61	0.42
**Affective health: anxiety**	SF-36 mental health	HADS anxiety	0.54 vs 0.35	< 0.001	0.66 vs 0.45	< 0.001	0.72	0.47
**Affective health: anxiety**	MPI negative mood	HADS anxiety	0.61 vs 0.35	< 0.001	0.65 vs 0.45	< 0.001	0.68	0.37
**Coping (control)**	MPI control pain	CSQ control pain	0.46 vs 0.30	0.011	0.44 vs 0.29	0.011	0.60	0.33

NRS: Numeric Rating Scale, SF-36: Short Form 36, MPI: Multidimensional Pain Inventory, HADS: Hospital Anxiety and Depression Scale, CSQ: Coping Strategies Questionnaire. ES: Effect Size, SRM: Standardized Response Mean. p: Type I error of the modified Jacknife test comparing two ES or two SRM. r base: bivariate Spearman rank correlation of the baseline scores, r diff: bivariate Spearman rank correlation of the effects (raw score differences baseline → follow-up)

The correlation coefficients of the baseline scores and raw score differences, i.e. absolute effects (Table 2, 2^nd ^from last and last columns) for the scales under comparison showed moderately till highly (correlation coefficient > 0.70) overlapping constructs. The highest values reflecting good convergence were found for the affective health comparisons with values of up to 0.73 and for pain with values of up to 0.76. In the function and the physical role interference comparisons, only the comparison of MPI interference with SF-36 social functioning showed moderate correlations (up 0.50). The SF-36 physical functioning did not correlate with the MPI activity showing divergent/discriminant constructs.

## Discussion

We examined the ability of five self-assessed outcome instruments to sensitively measure changes in physical and mental health and quality-of-life in 273 chronic pain patients before and after a four week inpatient interdisciplinary pain program. The pain-specific MPI, specially developed for chronic pain conditions, was most responsive – or at least equally responsive as the compared scales – in the domains of pain, role and social interference with pain, depression, anxiety, and control of pain. The SF-36 was equally or more responsive than the pain NRS, the mood-specific HADS, and the coping-specific CSQ in the comparable domains. Both, HADS and CSQ are to some extent also generic measures as being applicable to various health and behavioural conditions. Besides interference with pain, responsiveness in function cannot be compared due to divergent constructs – the MPI asks about very specific functions in its activity dimension whereas the SF-36 covers physical tasks more generally, e.g. ambulation. Overall, the correlation data showed moderate (to partly high) overlap of the constructs of the compared scales in pain, coping, and affective health and were comparable to those reported previously between the SF-36 and the MPI [19].

The MPI confirmed our hypothesis that a condition-specific instrument measures more responsively than a generic one (the SF-36). The mood-specific HADS and the coping-specific CSQ failed to fulfil this hypothesis as far as could be determined on the basis of the compared scores. In other words, the generic SF-36 (also) is an excellent tool in the assessment of affective health, social function and role performance (physical and emotional) in chronic pain.

All examined domains of health and quality-of-life are important for the patient and are particularly addressed by treatment management in accordance with the International Classification of Functioning, Disability and Health – ICF [37]: In most cases chronic pain can not be eradicated but the patient can learn to tolerate, manage, and compensate for its consequences [38]. Most of the effect differences were small, a typical characteristic or problem in the measurement of chronic conditions, whether changes in health are due to the natural course of the disorder or due to treatment interventions. As a consequence, in clinical and interventional studies, it is essential to use a control group design and choose the most responsive instrument for the detection of changes in outcome in order to keep the sample size – and by that the costs – low. Vice versa, observed effects may remain not significantly different from zero or from the effect of a control group when measured by less responsive instruments.

In addition, the importance of effects which are smaller than the MID or the MCID is questionable since they are (on average) below the level of what the patients can perceive as a meaningful change. A more responsive instrument is able to measure higher effects above the MCID than an instrument with low responsiveness. To estimate MCID by the SEM is notably much more conservative than to determine the MCID by the "transition method", which asks the patients directly to rate their global health change (the anchor) and which relates this assessment to the measured size of ES or SRM [35,39]. For example, for the Western Ontario and McMaster Universities osteoarthritis index (WOMAC) global score, the MCID estimated by the SEM was 0.54 in ES units, whereas the MCID by transition method resulted in 0.40 [35].

The choice of the responsiveness parameter depends on the focus of interest and the characteristics of the different methods as outlined in the background. For the decision between ES and SRM, there is a directive rule [40]. The following scales showed pre-treatment to post-treatment scores rank correlations lower than 0.50 (data not shown in detail): Pain NRS, SF-36 role physical, bodily pain, social functioning, role emotional, MPI pain severity, life control, control pain, CSQ control pain and decrease pain. As parameter for responsiveness for these scales, the ES would be more appropriate than the SRM; for all other scales with correlation ≥ 0.50, it would be the SRM [40].

To our knowledge and after an extensive search in MedLine, there is only one comparable study which assessed the responsiveness of the MPI and the SF-36 in chronic pain patients referred to an outpatient interdisciplinary pain program [19]. The SRMs between the MPI and the SF-36 were not significantly different within the domains (t-test): MPI pain severity versus SF-36 bodily pain, 0.41 versus 0.44; MPI interference with pain versus SF-36 social functioning, 0.42 versus 0.25; MPI negative mood versus SF-36 mental health, 0.22 versus 0.09. The comparisons MPI interference with pain versus SF-36 role physical, 0.42 versus 0.03, and MPI activity versus SF-36 physical functioning, 0.22 versus 0.27, were not statistically tested since these domains showed a small overlap and correlation in the regression analysis whereas the other three (pain, interference, mood) largely overlapped – our data reproduced these overlap findings. In summary, these results were only partly consistent with our results but failed significance was probably caused by the small sample size (n = 87), the less sensitive t-test compared to modified Jacknife test, and small effects that were often below the MCID. Another study used some scales of the SF-36, MPI, and CSQ in 142 FM patients after a multidisciplinary outpatient pain program [41]. Out of these data, high ES can be determined ranging from 0.84 to 1.79. However, the only possible comparison is that between MPI interference (ES = 1.65) and SF-36 role physical (ES = 1.79) which is not significantly different (t-test) and is consistent to our findings.

The study's strengths are the large, prospectively examined cohort with consistent characteristics and almost no missing data. The assessment instruments used are well known worldwide, profoundly tested, and permit standardized measurement and comparison between cohorts of different conditions, countries, and cultures. To our knowledge, there is no previously published study which compared the five instruments for chronic pain. As a limitation it must be stated that the transition question to determine the MCID more precisely was not asked but this does not affect the responsiveness comparison itself [35,39].

## Conclusion

The pain-specific MPI was most responsive in all comparable domains followed by the generic SF-36. Both can be recommended for comprehensive and specific bio-psycho-social effect measurement of health and quality-of-life in chronic pain.

## Competing interests

The authors declare that they have no competing interests.

## Authors' contributions

All authors commented on the draft and the interpretation of the findings, helped to write and approved the final manuscript. FA was responsible for all parts of the work of the study, especially for the analysis and the interpretation of the data, and wrote the original manuscript. MLV helped in the data analysis, contributed to the design of the study, the interpretation of the findings, their presentation, and the writing of the manuscript. SL was responsible for the data acquision, helped in the analysis and the interpretation of the data. AA was responsible for the conception, the design, and the resources for the study and helped to prepare the manuscript.

## Pre-publication history

The pre-publication history for this paper can be accessed here:


